# Development of Dementia Best Care Practices in Long-Term Care Settings Online Modules

**DOI:** 10.1093/geroni/igaa057.1720

**Published:** 2020-12-16

**Authors:** Kara Dassel, Larry Garrett, Troy Andersen, James Ballard, Martin Freimer, Jorie Butler, Linda Edelman

**Affiliations:** 1 University of Utah, Salt Lake City, Utah, United States; 2 University of Utah - College of Nursing, Salt Lake City, Utah, United States

## Abstract

One arm of the Utah Geriatrics Education Consortium focuses on providing Alzheimer’s disease and related dementias (ADRD) education to formal and informal caregivers. An interdisciplinary group of faculty developed a 3-hour online training program focused on best care practices, recommended by the National Alzheimer’s Association, for persons with ADRD residing in long-term care settings. Topic include: 1) Overview of Dementia (e.g., What are types of dementia and the associated symptoms?), 2) Understanding Behaviors and Your Approach (e.g., What are the best methods for recognizing and reporting behavior change?), 3) Effective Communication within Long-Term Care (e.g., What are the best strategies to enact effective team meetings?), and 4) Communication and Responding to Behaviors (e.g., What are methods for reducing patient agitation?). Throughout the four modules a case study follows “Mrs. Jones” to demonstrate the skills and techniques raised in each module. Pre- and post-evaluation questions are embedded in the training program.

